# Two Adenine Nucleotide Translocase Paralogues Involved in Cell Proliferation and Spermatogenesis in the Silkworm *Bombyx mori*

**DOI:** 10.1371/journal.pone.0119429

**Published:** 2015-03-05

**Authors:** Ryohei Sugahara, Akiya Jouraku, Takayo Nakakura, Takahiro Kusakabe, Takenori Yamamoto, Yasuo Shinohara, Hideto Miyoshi, Takahiro Shiotsuki

**Affiliations:** 1 Insect Growth Regulation Research Unit, National Institute of Agrobiological Sciences, Tsukuba, Ibaraki, Japan; 2 Insect Genome Research Unit, National Institute of Agrobiological Sciences, Tsukuba, Ibaraki, Japan; 3 Laboratory of Silkworm Science, Kyushu University Graduate School of Bioresource and Bioenvironmental Sciences, Fukuoka, Japan; 4 Institute for Genome Research, University of Tokushima, Tokushima, Japan; 5 Division of Applied Life Sciences, Graduate School of Agriculture, Kyoto University, Kyoto, Japan; Ecole Normale Superieure de Lyon, FRANCE

## Abstract

Mitochondrial adenine nucleotide translocase (ANT) specifically acts in ADP/ATP exchange through the mitochondrial inner membrane. This transporter protein thereby plays a significant role in energy metabolism in eukaryotic cells. Most mammals have four paralogous *ANT* genes (*ANT1-4*) and utilize these paralogues in different types of cells. The fourth paralogue of *ANT* (*ANT4*) is present only in mammals and reptiles and is exclusively expressed in testicular germ cells where it is required for meiotic progression in the spermatocytes. Here, we report that silkworms harbor two ANT paralogues, the homeostatic paralogue (BmANTI1) and the testis-specific paralogue (BmANTI2). The BmANTI2 protein has an N-terminal extension in which the positions of lysine residues in the amino acid sequence are distributed as in human ANT4. An expression analysis showed that BmANTI2 transcripts were restricted to the testis, suggesting the protein has a role in the progression of spermatogenesis. By contrast, BmANTI1 was expressed in all tissues tested, suggesting it has an important role in homeostasis. We also observed that cultured silkworm cells required BmANTI1 for proliferation. The ANTI1 protein of the lepidopteran *Plutella xylostella* (PxANTI1), but not those of other insect species (or PxANTI2), restored cell proliferation in BmANTI1-knockdown cells suggesting that ANTI1 has similar energy metabolism functions across the Lepidoptera. Our results suggest that BmANTI2 is evolutionarily divergent from BmANTI1 and has developed a specific role in spermatogenesis similar to that of mammalian ANT4.

## Introduction

To fulfill the high energy demands for physically strenuous activities such as flight and for developmental morphogenesis, insects have evolved a complex system controlling energy metabolism. The insect fat body is one of the main centers of energy storage and utilization and displays considerable biosynthetic and metabolic activities [1]. In addition, insects that undertake long-distance flight appear to have evolved an increased number of genes involved in metabolizing their fuel source [2]. Silkworms, a well-established insect model, have been found to possess a unique metabolic pathway for energy supply to the spermatozoa [3].

Adenine nucleotide translocase (*ANT*) genes, also known as ADP/ATP carrier genes (*AAC*), encode membrane proteins that participate in the exchange of ADP and ATP across the inner mitochondrial membrane and thus play a substantial role in cell energy metabolism [4,5]. These proteins are members of the mitochondrial carrier protein family and share a similar structure consisting of three homologous repeat domains that contributes to their activities in transport across the inner mitochondrial membrane [6,7]. Among the mitochondrial carrier protein family, only ANTs have the signature amino acid sequence RRRMMM, which is critical for ATP/ADP transport activity [8,9].

Currently, four human *ANT* paralogues have been identified (*HsANT1*, *2*, *3*, and *4*) and their expression profiles have been reported for various tissues and cultured cells: *HsANT1* is mainly expressed in heart and skeletal muscle [10,11]; *HsANT2* is expressed in proliferative cells and appears to be required for glycolysis [12,13]; *HsANT3* is ubiquitously expressed in many tissues [14] although, interestingly, rodents have lost this paralogue during evolution; *HsANT4* is exclusively expressed in testicular germ cells [15]. The latter gene was initially thought to be a mammalian-specific paralogue but has recently also been identified in the green anole lizard [16]. The HsANT4 protein has a similar amino acid sequence to ANT1-3 (66–68% identity); however it includes an N-terminal region in which several charged residues, such as lysine, are present [15]. In addition to differences in the N-terminus, the C-terminus of HsANT4 has an extension of a few residues [15]. Interestingly, expression of mouse *ANT4* rises in preleptotene spermatocytes, peaks at early pachytene, and then decreases at late pachytene and in round spermatids [17]. Consistent with this expression pattern, *Ant4*-deficient mice exhibit disruption to meiosis in the testis during the early stages of meiotic prophase I, suggesting that the protein is required for a continuous supply of large amounts of ATP to meiotic cells [18]. There is also evidence that HsANT4 co-localizes with glycolytic enzymes in the principal piece of the sperm flagellum, suggesting it has a role in sperm motility [19].

The AAC proteins of *Saccharomyces cerevisiae* are encoded by three paralogous genes, *ScAAC1* to *3*. The expression of each gene in yeast cells depends on the presence/absence of a fermentable carbon source and oxygen [20–23], suggesting that *S*. *cerevisiae* utilizes different AAC paralogues in order to overcome variations in external nutrient and oxygen conditions. Heterologous expression of HsANT1, 2 or 3 proteins or of HsANT4 with a point mutation restores respiration in a yeast strain that lacks the three endogenous AAC paralogues, and enables the yeast to grow on a non-fermentable carbon source [24–27].

*Drosophila melanogaster* harbors two ANT proteins that are generated by alternative splicing. They appear to be transcribed from a common promoter [28]. DmAnT1 was originally identified in a stress-sensitive mutant (sesB; CG16944) that showed conditional paralysis in response to a mechanical stress [28,29]. *DmAnt1* null alleles are lethal and knockdown or overexpression also results in developmental lethality [30], indicating the crucial role of the protein in cellular energy metabolism during development. The function of DmANT2 (Ant2: CG1683) has yet to be fully resolved.

ANT proteins have been under investigation for a considerable time and it has been found that their expression is strictly controlled in tissue-dependent and condition-dependent manners [11,15,21,31,32]. Most eukaryotes have multiple ANT proteins that have high amino acid similarities, although the patterns of possession of the paralogues differ even among vertebrates [16]. It is currently unclear what factors determine which paralogues will be present in any given organism. Functional characterization of ANT paralogues across a range of organisms will undoubtedly provide insights into this question. Here, we report that the lepidopteran species *Bombyx mori* has two ANT paralogues and that *Plutella xylostella* has at least three paralogues. Lepidopteran ANTI2 has a similar amino acid sequence to mammalian ANT4. Our data also showed, in the silkworm, that the other ANT (BmANTI1) was found to be essential for cell proliferation in cell cultures. Decreased proliferation in BmANTI1-knockdown cells was restored by ectopic expression of *P*. *xylostella* ANTI1 but not by any ANT paralogue of insects of other orders. These results suggest that Lepidoptera share common energy metabolism functions, and that these differ in insects of other orders. The specific requirements of lepidopteran energy metabolism that might underlie this difference are discussed in this paper.

## Materials and Methods

### Identification and cloning of *ANT* genes

Total RNAs were extracted using ISOGEN (Nippon Gene) and the SV Total RNA Isolation System (Promega) from the whole body of laboratory colonies of silkworm strain C145xN140 male larvae of 5th instar (*B*. *mori*), several stages of *D*. *melanogaster* Canton-S strain (larvae, pupae, and adults), last instar larvae of diamondback moth (*P*. *xylostella*), last instar larvae of smaller tea tortrix (*Adoxophyes honmai*), *Bemisia tabaci* Biotype-Q at day 0 of adults, gregarious 3rd nymphs (*Schistocerca gregaria*) [33], *Nilaparvata lugens* strain Izumo of 3rd nymphs, and *Tetranychus urticae* Kock (green form) from G1 population in the previous report [34], and from the whole body of *Stenotus rubrovittatus* collected from experimental paddy field of Agricultural Research Center of National Agriculture and Food Research Organization at Ibaraki Prefecture by its staff researcher. All insects used in this study were not endangered or protected species. Each RNA was converted into cDNA using Superscript III and oligo(dT) primer (Invitrogen). *ANT* gene open reading frames (ORFs) were identified in cDNA databases of the National Center for Biotechnology Information, the National Institute of Agrobiological Sciences, Bioinformatics & Evolutionary Genomics (http://bioinformatics.psb.ugent.be/), and the sequencing data from our RNA-seq analysis in the present study. The full-length ORFs of *SrANTI2*, *SrANTI3 NlANTI1*, *NlANTI2*, and *TuANT* were determined by 5’ and 3’ rapid amplification of cDNA ends using a GeneRacer kit (Invitrogen). The 5’ ends of the *ANT* ORFs were subcloned and sequenced to confirm whether they were correctly predicted. The full-length ORFs of *ANT* genes were amplified using the primer pairs listed in S1 Table and inserted into a pENTR11 (Invitrogen, Life technologies) vector, and their nucleotide sequences were determined via dye-terminator cycle sequencing using a DNA sequencer 3130 (Applied Biosystems).

### RNA-seq analysis

Total RNAs of *S*. *gregaria*, *S*. *rubrovittatus*, and *B*. *tabaci* were extracted using ISOGEN from the whole body of gregarious 3rd nymphs, the adult whole body collected from paddy field, and the whole body of *B*. *tabaci* Biotype-Q at day 0 of adults, respectively, and purified using the SV Total RNA Isolation System. Preparation of cDNA libraries from the total RNAs and sequencing by Illumina HiSeq 2000 sequencer were performed by Hokkaido System Science Co., Ltd. (Sapporo, Japan). RNA-seq reads of *S*. *gregaria*, *S*. *rubrovittatus*, and *B*. *tabaci* were *de novo* assembled by Trinity and 64921, 70502, and 62096 contigs were generated.

### Nucleotide sequence submission

The Nucleotide and amino acid sequences identified in the present study have been submitted to the DDBJ (BmANTI1; AB928002, BmANTI2; AB928003, PxANTI1; AB928004, PxANTI2; AB928005, PxANTI3; AB928006, AhANTI1; AB928007, AhANTI2; AB928008, SrANTI1; AB928009, SrANTI2; AB928010, SrANTI3; AB928011, BtANTI1; AB928012, SgANTI1; AB928013, SgANTI2; AB928014, NlANTI1; AB928015, NlANTI2; AB928016, and TuANT; AB928017). The obtained RNA-seq data of *S*. *gregaria*, *S*. *rubrovittatus*, and *B*. *tabaci* have been deposited in DRA under accession number DRA002231.

### Phylogenetic analysis

Amino acid sequences of the insect and vertebrate ANT genes were aligned using the CLUSTAL-W program, and the phylogenetic tree was constructed with the GENETYX software Version 11.0 (Genetyx) using the neighbor-joining method (bootstrap trials, 1000 times; TOSSGAPS, on).

### RNA isolation from *B*. *mori*

In order to obtain whole-body RNA from different stage of *B*. *mori*, silkworm race C145xN140 was reared as described previously [35]. A series of RNAs were extracted from day 3, 2, 2, 2, 4, and 4 of the embryo stage, 1st, 2nd, 3rd, 4th, and 5th instar larvae, and adult stage, respectively. The numbers of individuals used were 40, 10, 5, 3, 1, and 1 at each stage. For tissue samples of larvae, silkworm race Ariake was reared as well as C145xN140. The 4th and 5th instars of the male Ariake were dissected, and tissues were separated. The numbers of individuals used for tissue samples were 3 and 2/day for the 4th and 5th instar larvae, respectively. Total RNA was extracted using ISOGEN and purified using the SV Total RNA Isolation System.

### Semi-quantitative reverse transcription-PCR (semi-qRT-PCR) and quantitative reverse transcription-PCR (qRT-PCR) analysis

The first strand of cDNA was synthesized from the isolated total RNA using SuperScript III reverse transcriptase (Invitrogen) and oligo(dT) primer according to the manufacture’s instructions. Semi-qRT-PCR amplifications were performed to largely evaluate expression levels of the target genes. For qRT-PCR analysis, serial dilutions of pENTR-BmANTI1 and pENTR-BmANTI2 plasmids were used as standards. *B*. *mori* ribosomal protein 49 (*Bmrp49*) was used as a reference gene. The template plasmids and primers for *Bmrp49* were prepared as described previously [36]. qRT-PCR primer pairs for *BmANTI1* and *BmANTI2* were listed in S1 Table. qRT-PCR data were obtained following the previous procedure [37]. The molar amounts of transcripts of targets were calculated based on crossing point analysis, using standard curves generated from the plasmids standards. *BmANTI1* and *BmANTI2* transcript levels were normalized with *rp49* transcript levels in the same samples.

### Cell culture and transfection

BmN4-SID1, the previously established cell line that expressed a *Caenorhabditis elegans* SID-1 protein in order to take up double-stranded RNA into silkworm cells [38], were maintained at 27°C in EX-CELL 420 medium (Sigma) supplemented with 10% fetal bovine serum (Biowest). Expression vectors were transfected into the silkworm cells using Fugene HD (Promega) during overnight incubation. After replacement of the medium with new EX-CELL 420 medium, the cells were incubated for 3 days.

### Subcellular localization analysis

The entry clones of the *ANT* genes in *B*. *mori*, *P*. *xylostella*, *S*. *gregaria*, *N*. *lugens*, *D*. *melanogaster*, and *T*. *urticae* were transferred into the expression vector of pie2GW by gateway reaction to construct the plasmids expressing recombinant proteins with N-terminal GFP fusions [39]. After transfection of the resulting expression constructs, BmN4-SID1 cells expressing ANTs fused to GFP were seeded on poly-L-lysine-coated coverslips (Matsunami) and incubated overnight. For mitochondrial staining, cells on coverslips were incubated with 200 nM MitoTracker Red CMXRos (Molecular Probes) for 30 min at 27°C in culture medium. Then, cells were washed three times with phosphate buffered saline (PBS) and fixed with 4% paraformaldehyde at room temperature for 10 min. A series of images were acquired using a Zeiss LSM 700 confocal microscope.

### Mitochondria isolation

Pellets of BmN4-SID1 cells expressing target proteins from 25 cm^2^ culture plate were harvested and washed with phosphate-buffered saline (PBS). One-fifth of the pellet was lysed in PBS by sonication, and the supernatant was retrieved after centrifugation. From the remaining pellet, mitochondria were isolated using a Mitochondria Isolation Kit for Cultured Cells (Pierce) according to the manufacture’s protocol, resulting in obtaining 130 μl cytosolic fraction and 100 μl mitochondrial fraction. Five microliters of each of these fractions were subjected to Western blotting using anti-FLAG antibody (M2, Sigma) or anti-α-tubulin antibody (Abcam; ab7291). The presence of α-tubulin protein was confirmed to verify that mitochondrial fraction did not include contaminants.

### Cell proliferation assay

Cell proliferation assays were carried out using Cell Counting Kit-8 (Dojindo) by a similar method as described previously [40]. In brief, BmN4-SID1 cells were soaked with double-stranded RNAs for BmANTI1 to induce gene silencing. Three days after incubation with the dsRNAs, cells were seeded onto microtiter plates at a density of 2.4 x 10^4^, 1.2 x 10^4^, 0.6 x 10^4^, and 0.1 x 10^4^ cells per well with culture medium containing additional dsRNAs. After incubation for four, seven, nine, or fourteen days at 27°C, WST-8 solution was added to each well and an absorbance of each well was taken as a measure of living cells. The cell proliferation of BmANTI1-knockdown cells relative to cells untreated with dsRNA was plotted. Statistical significance was evaluated by the Student’s *t*-test, and a *P*-value <0.01 was considered statistically significant.

### Establishment of cell lines stably expressing ANT proteins

Expression cassettes for *FLAG*-tagged *ANT* cDNA under the control of ie2 promoter were cloned into pPG132, a *piggyBac*-based transposition vector, using the Gateway system [41]. BmN4 cells were co-transfected with the pPG132-*ANT* and helper plasmid encoding *piggyBac* transposase, resulting in selection of the transformed cells as described previously [42].

### Double-stranded RNAs

Three and six cDNA fragments for the *BmANTI1* gene and the *TcANT* genes were amplified from total RNA of the silkworm BmN4 cells and *Tribolium castaneum* individuals by RT-PCR using the primers listed in S1 Table and cloned into pLits vector [42]. Based on these plasmids, nine dsRNAs were generated by *in vitro* transcription using MEGASCRIPT T7 transcription kit (Ambion) according to manufacture’s protocol [42].

### RNAi experiment in *T*. *castaneum*

*T*. *castaneum* used in this study was reared as described previously [43]. *Tribolium* larvae at 14 days after egg-laying were injected with the largest possible volume of 5 μg/μl dsRNA as described previously [43]. At three days post-injection, three larvae were sampled out of the injected individuals and integrated into one sample for each treatment, following which the RNAi efficiencies were verified by semi-qRT-PCR.

## Results

### Isolation of *ANT* genes and analysis of amino acid sequences

The nucleotide sequences of *Ant Insect* homologues were determined by a combination of computational prediction and RACE analyses for the following insect species: *P*. *xylostella*, *Stenotus rubrovittatus*, *Bemisia tabaci*, *Schistocerca gregaria*, *Nilaparvata lugens*, *Tetranychus urticae*, *B*. *mori*, and *Adoxophyes honmai*. Table 1 provides data on scientific classification of each arthropod species. Sequencing data from the transcriptomes of *S*. *rubrovittatus*, *B*. *tabaci* and *S*. *gregaria* were acquired using RNA-seq on total RNA (see Materials and methods). Full-length ORF clones were amplified and inserted into an entry vector for the Gateway system (Invitrogen). The *ANT* ORFs in the entry vector were sequenced and their amino acid sequences were predicted.

**Table 1:** A list of arthropod species examined.

Class	Order	Family	Species
Insecta	Lepidoptera	Bombycidae	*Bombyx mori*
		Nymphalidae	*Danaus plexippus*
		Plutellidae	*Plutella xylostella*
		Tortricidae	*Adoxophyes honmai*
	Diptera	Drosophilidae	*Drosophila melanogaster*
	Coleoptera	Tenebrionidae	*Tribolium castaneum*
	Hemiptera	Delphacidae	*Nilaparvata lugens*
		Aleyrodidae	*Bemisia tabaci*
		Miridae	*Stenotus rubrovittatus*
	Orthoptera	Acrididae	*Schistocerca gregaria*
Arachnida	Trombidiformes	Tetranychidae	*Tetranychus urticae*

**Fig 1:**
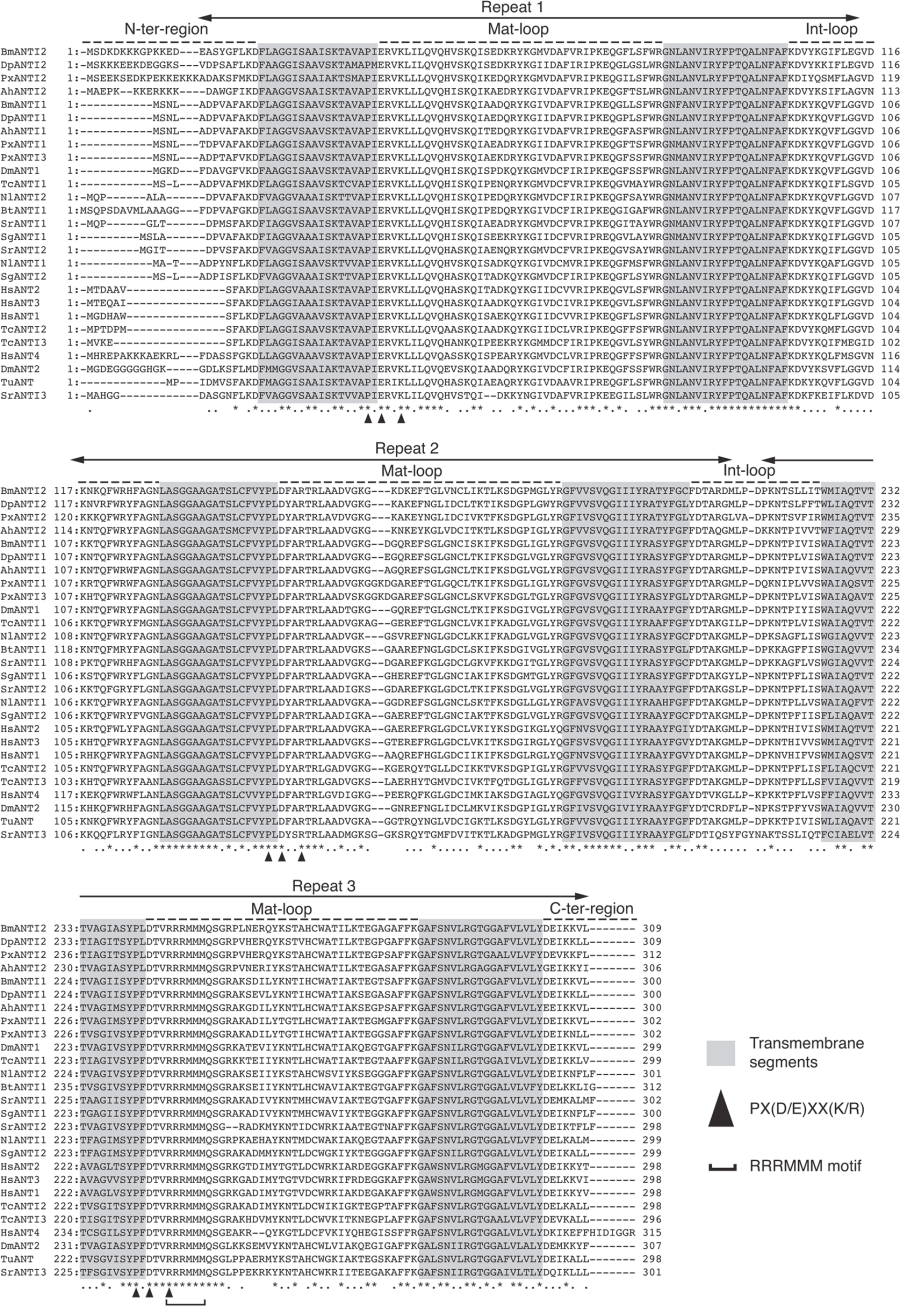
Amino acid sequences of insect ANT. Sequence alignment of the ANT proteins. The Genetyx software and CLUSTAL-W program were used for arrangement. Three homologous repeated domains are shown with arrows. The loops of the matrix side and intermembrane space side of mitochondria are indicated as mat-loop and int-loop on dashed line, respectively. Transmembrane segments located on the inner membrane of mitochondria are highlighted in light gray. Conserved residues marked by arrowheads represent the PX(D/E)XX(K/R) sequence. The RRRMMM signature is observed in all the ANT proteins aligned. HsANTI1 (NP_001142), HsANTI2 (NP_001143), HsANTI3 (NP_001627), HsANTI4 (NP_112581), DpANTI1 (EHJ74594), DpANTI2 (EHJ77067), DmANT1 (NP_727448), DmANT2 (NP_511110), TcANTI1 (XP_968561), TcANTI2 (XP_973257), and TcANTI3 (EFA07039) were retrieved from the NCBI/GenBank database. Abbreviations: Bm, *Bombyx mori*; Dp, *Danaus plexippus*; Px, *Plutella xylostella*; Ah, *Adoxophyes honmai*; Dm, *Drosophila melanogaster*; Tc, *Tribolium castaneum*; Nl, *Nilaparvata lugens*; Bt, *Bemisia tabaci*; Sr, *Stenotus rubrovittatus*; Sg, *Schistocerca gregaria*; Hs, *Homo sapience*; Tu, *Tetranychus urticae*.

Sequence comparisons of human, insect and mite ANTs showed the presence of high levels of homology over the entire amino acid sequence. HsANT1, for example, exhibited 80% and 75% identities with BmANTI1 and BmANTI2, respectively. BmANTI1 also showed similarity to its paralogue BmANTI2 (80% identity). All the ANT proteins examined had the ANT signature RRRMMM motif and a highly conserved structure consisting of three tandem repeats of the PX(D/E)XX(K/R) sequence, which is characteristic of mitochondrial carrier proteins (Fig. 1). Notably, the sequence alignment revealed an extra sequence of approximately 10 amino acids at the N-terminus in BmANTI2, AhANTI2, PxANTI2, DpANTI2, BtANTI1, DmANT2, and HsAnt4. In particular, the N-terminal regions of lepidopteran ANTI2 and of HsAnt4 were lysine rich, while many glycine residues were present in DmANT2. No C-terminal extensions were observed in insect or mite ANTs, unlike HsANT4.

**Fig 2:**
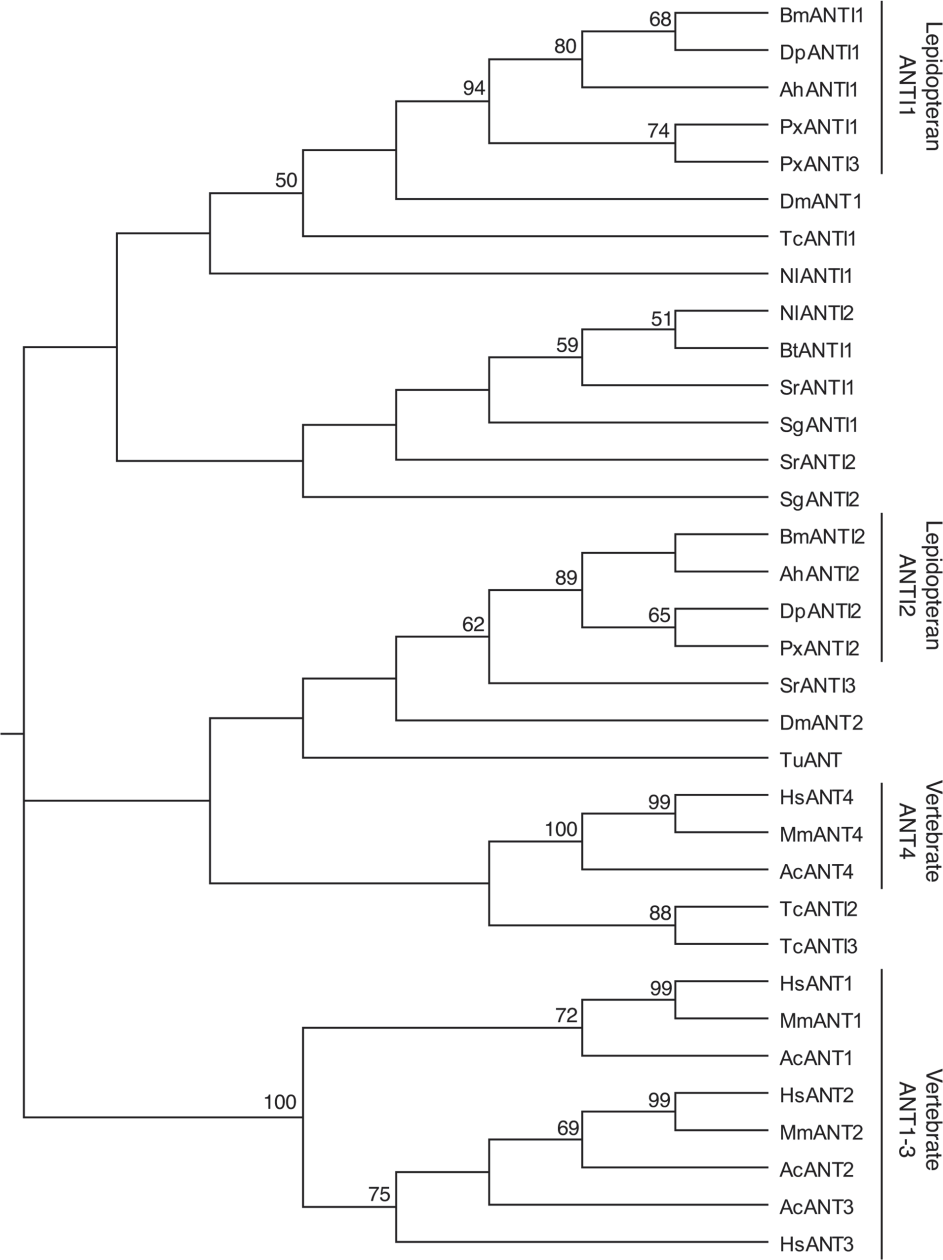
Phylogenetic tree of ANT proteins. Phylogenetic analysis of the ANT proteins. The neighbor-joining tree was generated in Genetyx software with the multiple sequence alignment. The numbers at the nodes denote bootstrap values (%). Bootstrap values <50% are not indicated. Sequences of mouse and anole lizard ANT genes, MsANT1 (NP_031476), MsANT2 (NP_031477), MsANT4 (NP_848473), AcANT1 (ENSACAP00000002268), AcANT2 (ENSACAP00000012600), AcANT3 (ENSACAP00000005895), and AcANT4 (ENSACAP00000011672) were retrieved from the NCBI/GenBank and Ensembl databases. Abbreviations: Mm, *Mus musculus*; Ac, *Anolis carolinensis*.

The phylogeny of the ANT proteins was investigated by constructing a phylogenetic tree of full-length amino acid sequences using the neighbor-joining method (Fig. 2). High bootstrap values were obtained at many nodes within vertebrate ANT proteins, but not within insect ANTs. However, lepidopteran ANTI2 proteins were distinguishable from lepidopteran ANTI1 lineage with relatively high bootstrap values, implying that the roles of BmANTI1 and BmANTI2 are conserved in each of the lepidopteran ANTI1 and ANTI2 linages, respectively.

### *BmANTI2* transcripts are specifically expressed in the testis

**Fig 3:**
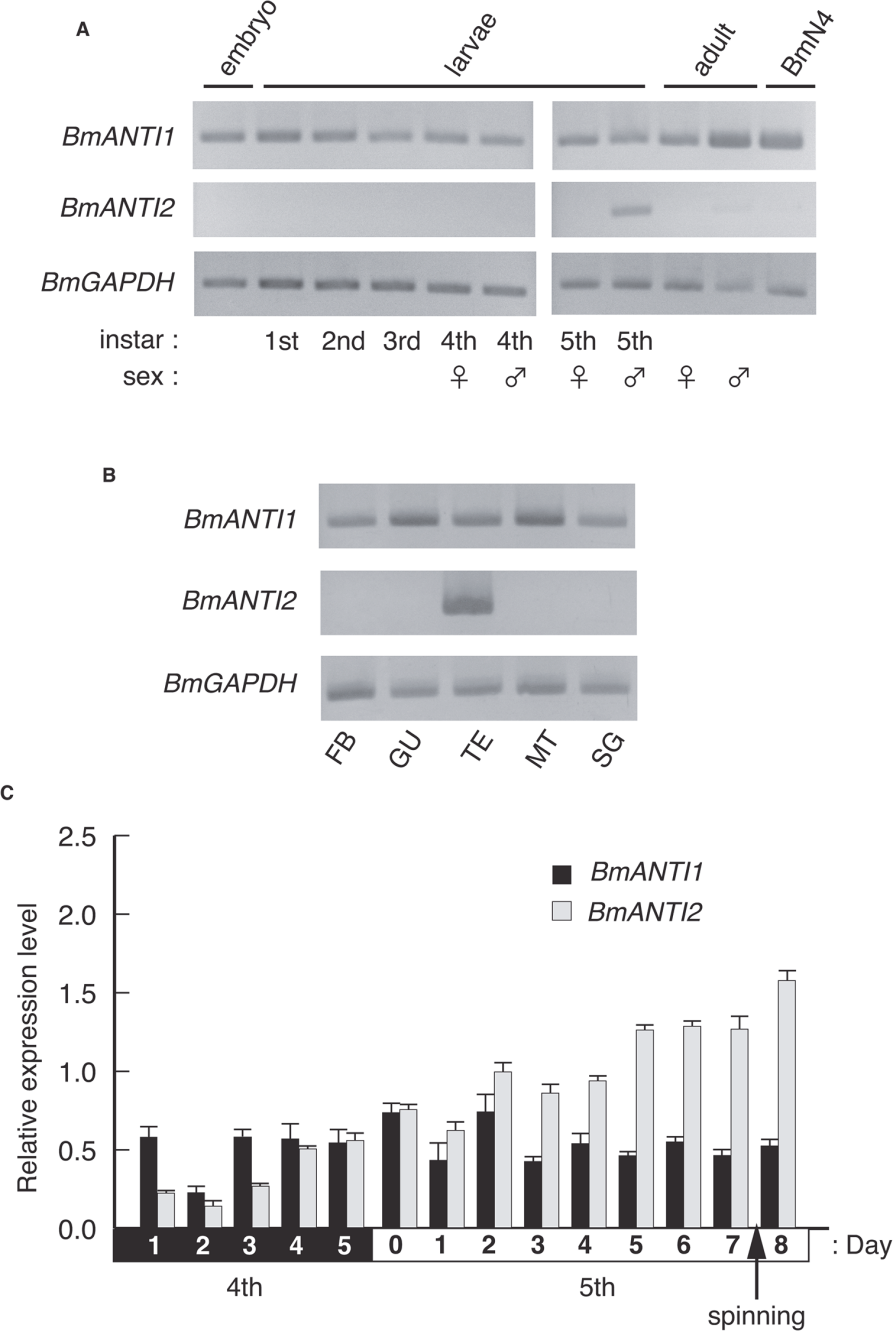
Expression profiles of *BmANTI1* and *BmANTI2* genes. The developmental and tissue-specific expression of *BmANT* genes. (A) Developmental expression profiles of *BmANTI1* and *BmANTI2* genes. Whole body RNA samples were extracted from embryo to adult, and subjected to semi-quantitative reverse transcription (semi-qRT)-PCR analysis. Total RNA from BmN4-SID1 cells was also included. Amplifications of GAPDH cDNA were used as an internal control. Individuals from 4th instar larvae to adult were divided into female and male. (B) Tissue expression profiles of *BmANTI1* and *BmANTI2* genes. The fat body (FB), gut (GU), testis (TE), Malpighian tubules (MT), and silk gland (SG) were retrieved from individuals at day 5 of 5th instar male larvae, and their total RNAs were subjected to semi-qRT-PCR analysis. (C) Testis expression profiles of *BmANTI1* and *BmANTI2* genes at the 4th and 5th instar larvae. The expression levels of *BmANT*s in the testis were measured by real-time PCR. Silkworms started to spin silk between day 7 and 8 of the 5th instar larvae. Relative expression levels against the *Bmrp49* gene in the testis are shown. Error bars represent the SD values of the means of triplicates.

The amino acid sequence analysis suggested that the two BmANTs had different features. We therefore compared their distribution patterns at different stages of development in *B*. *mori* by measuring mRNA expression levels in whole body samples using semi-quantitative PCR (Fig. 3A). We found that BmANTI1 was expressed consistently at all developmental stages and in BmN4 cells; by contrast, BmANTI2 expression was observed only in 5th instar male larvae. Next, we investigated whether the proteins were present in different tissues, namely, fat body, gut, testis, Malpighian tubules, and silk gland at day 5 in 5th instar stage male larvae. BmANTI1 was present in all tested tissues, whereas BmANTI2 was only present in the testis (Fig. 3B). These results indicate that *BmANTI1* and *BmANTI2* exhibit similar patterns of gene expression to mammalian *ANT2-3* and *ANT4*, respectively, suggesting that BmANTI2 might be an orthologue of human ANT4.

We examined the patterns of expression of BmANTI1 and BmANTI2 in the testis by quantitative reverse transcription (qRT)-PCR using testis cDNAs from 5th and 4th instar larvae. Relative expression levels were normalized against ribosomal protein 49 (*Bmrp49*). A relatively high level of expression of *BmAntI1* was present throughout the 4th and 5th instar stages (Fig. 3C). By contrast, the relative level of *BmANTI2* expression gradually increased from day 1 of the 4th instar stage to day 8 of the 5th instar stage, suggesting that the protein has an important role in spermatogenesis. It is noteworthy that *BmANTI2* was expressed in the testes of early of 4th instar stage larvae in which most germ cells are at early meiotic prophase.

### SgANTI1 and SgANTI2 are not testis-specific paralogues

**Fig 4:**
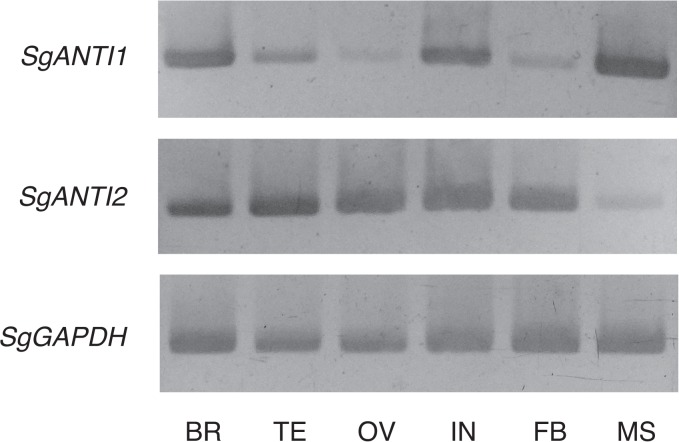
Tissue expression profiles of *SgANTI1* and *SgANTI2* genes. Brain (BR), testis (TE), ovary (OV), thoracic integument (IN), fat body (FB), and muscle (MS) were retrieved from desert locusts at day 1 of 3th instar nymphs, and their total RNAs were subjected to semi-qRT-PCR analysis. Amplifications of GAPDH cDNA were used as an internal control.

To test whether the testis-specific paralogue of *ANT* is conserved in a wide range of insect species, we investigated expression of *SgANTI1* and *SgANTI2* in several tissues, namely, brain, testis, ovary, thoracic integument, fat body, and muscle at day 1 in 3rd instar nymphs of the desert locust (Fig. 4). Semi-qRT-PCR analysis revealed that both *SgANTI1* and *SgANTI2* mRNA were present in all tested tissues. In particular, *SgANTI1* was strongly expressed in muscle tissue, where a high level of *ANT1* expression is detected in human, whereas expression of *SgANTI2* in muscle was very low compared to that in other tissues. These results suggest that *BmANTI2* is not functionally orthologous to *SgANTI1* nor *SgANTI2*. However, it remains a possibility that the desert locust possesses the testis-specific paralogue that is not identified.

### BmANTI1 is required for cellular proliferation in cultured silkworm cells

**Fig 5:**
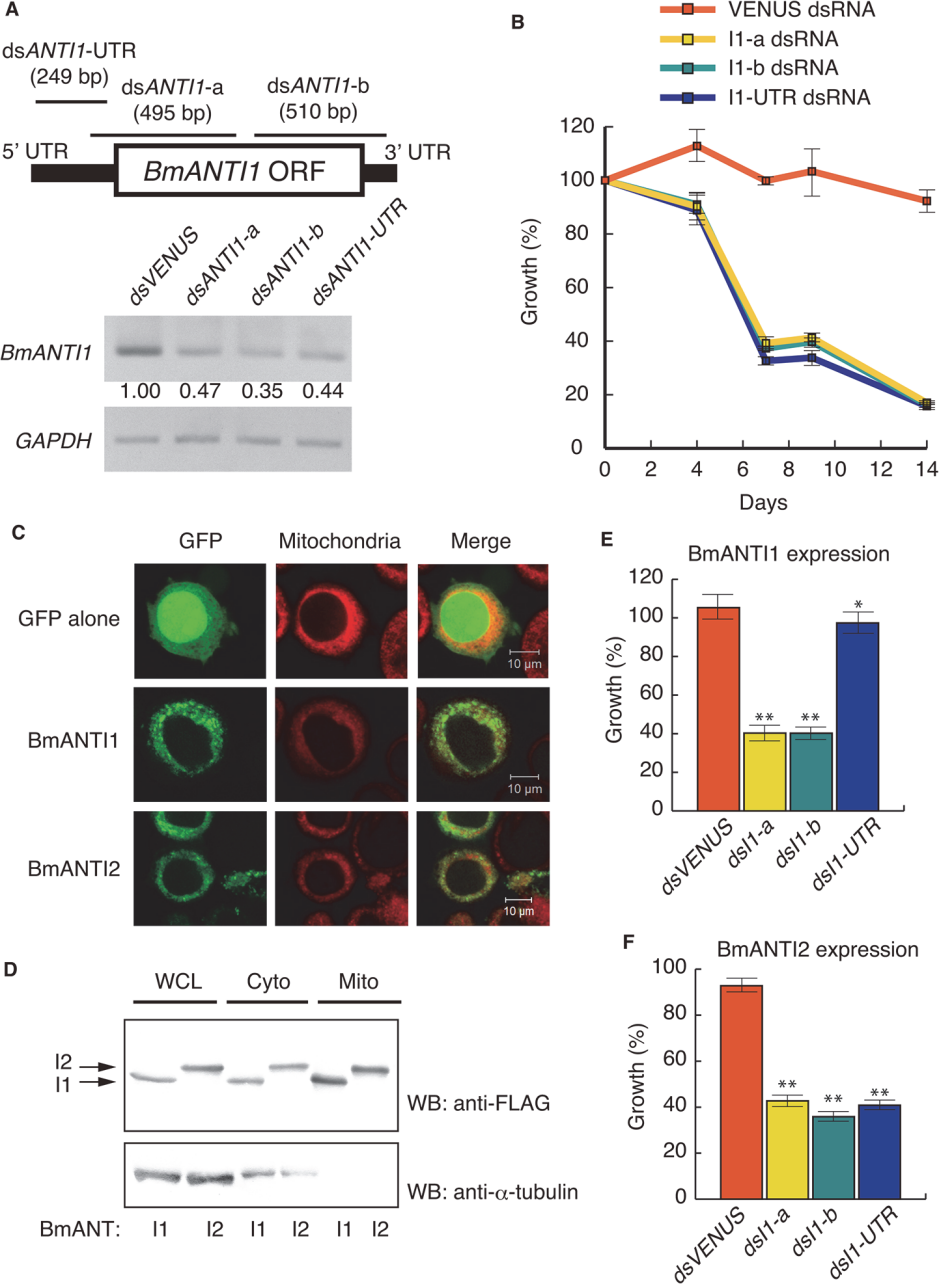
Requirement of the BmANTI1 for cell proliferation of BmN4-SID1 cells. BmANTI1 is required for cell proliferation in BmN4-SID1 cells. (A) Double-stranded (ds)RNA mediated gene silencing of BmANTI1 mRNA. To confirm the knockdown efficiency of dsRNAs on BmANTI1, semi-qRT-PCR analysis was performed. Lines on a schematic diagram of BmANTI1 represent three relative positions of dsRNA-targeted regions. The length of dsRNAs is shown in parentheses. PCR amplifications were carried out on cDNAs obtained from BmN4-SID1 cells soaked in VENUS (green fluorescent protein variant), BmANTI1-a, BmANTI1-b, and BmANTI1-UTR dsRNAs for 3 days. VENUS was used as a negative control that is unrelated sequence to silkworm genome. Transcript levels of the BmANTI1 gene were quantitated by IMAGEJ software. Amplifications of GAPDH cDNA were used as an internal control. (B) BmANTI1 depletion inhibits cell proliferation of BmN4-SID1 cells. Cell proliferations of BmANTI1 knockdown cells were assessed after 4, 7, 9 and 14 days culture. The data represent the percent growth as compared with dsRNA-untreated cells. Data are from one of four independent experiments with similar results. Error bars represent the SD values of the means of triplicate wells. (C) Mitochondrial localization of GFP-fused BmANTI1 and BmANTI2 in BmN4-SID1 cells. GFP alone or each GFP-fused BmANTI1 and BmANTI2 (green) was transiently expressed in BmN4-SID1 cells, and the subcellular localizations of these constructs were observed using confocal microscope. Mitochondria in cells were labeled with MitoTracher (red). (D) BmANTI1 and BmANTI2 stably expressed in BmN4-SID1 cells were efficiently transported to mitochondria. FLAG-tagged BmANTI1 or BmANTI2 was stably expressed in BmN4-SID1 cells, and the cells were fractionated into cytosolic (Cyto) and mitochondrial (Mito) compartments. Whole-cell lysates (WCL) were included to confirm protein expression. Each fraction was immunoblotted with anti-FLAG M2 and anti-α-tubulin antibodies. (E) Decreased cell proliferation of BmN4-SID1 cells depleted of endogenous BmANTI1 can be restored by expression of FLAG-tagged BmANTI1. Endogenous BmANTI1 was silenced by dsRNAs in BmN4-SID1 cells stably expressing FLAG-tagged BmANTI1. After 7 days incubation, the cells were subjected to cell proliferation experiment as described in (B). Data are from one of four independent experiments with similar results. Error bars represent the SD values of the means of triplicate wells. Differences in cell proliferation rate between BmANTI1-knockdown cells and cells soaked in VENUS dsRNA were evaluated with a two-tailed Student’s *t*-test. (*P < 0.05; **P < 0.01) (F) Expression of FLAG-tagged BmANTI2 fails to restore decreased cell proliferation of BmN4-SID1 cells depleted of endogenous BmANTI1. Using BmN4-SID1 cells stably expressing FLAG-tagged BmANTI2, cell proliferation assay was performed as described in (E). Data are from one of four independent experiments with similar results. Error bars represent the SD values of the means of triplicate wells. (**P < 0.01).

The silkworm cell line BmN4-SID1 is an RNAi-sensitive line that was generated from the widely used BmN4 cell line [38]. Analysis of gene expression profiles showed that *BmAntI1*, but not *BmAntI2*, was expressed in BmN4-SID1 cells (Fig. 3A). The ubiquitous expression of *BmAntI1* suggests that it has a fundamental role in cellular energy metabolism. To test whether BmANTI1 had a role in cell proliferation, we performed an RNAi knockdown in BmN4-SID1 cells using three dsRNAs that targeted *BmAntI1* mRNA (Fig. 5A). A dsRNA corresponding to the green fluorescent protein variant Venus was used as the negative control. The dsRNAs, ds*AntI1*-a and ds*AntI1*-b, corresponded to parts of the untranslated region (UTR) and open reading frame (ORF), while dsANTI1-UTR targeted part of the 5’-UTR. We found that each dsRNA induced a reduction in the levels of *BmAntI1* mRNA.

We then performed a proliferation assay in cells depleted of BmANTI1 using these dsRNAs (see Materials and methods). The cell proliferation curve of cells treated with each of the dsRNAs against *AntI1* showed a significant reduction in total cell number compared to cells treated with the *dsVENUS* negative control (Fig. 5B). Thus, silencing of *AntI1* clearly inhibited cellular proliferation.

### BmANT-GFP proteins localize to mitochondria in BmN4-SID1 cells

As described above, *BmAntI2* was not expressed in BmN4-SID1 cells. We next assessed whether expression of *BmAntI1* or *BmAntI2* could rescue the inhibition of cell proliferation induced by BmANTI1 knockdown. It is known that functional expression of heterologous human ANT proteins in yeast cells requires a sufficiently high level of expression of Ants and the delivery of the proteins to the mitochondria. Therefore, we first confirmed that BmANTI1 and BmANTI2 recombinant proteins localized to the mitochondria in BmN4-SID1 cells. We constructed plasmids expressing GFP-BmANTI1 or -BmANTI2 fusion proteins and transfected them into BmN4-SID1 cells. GFP alone was also expressed in the silkworm cells as a control. Fluorescence microscopic observation of the cells after MitoTracker Red staining showed mitochondrial localization of BmANTI1 and BmANTI2 (Fig. 5C), demonstrating that these recombinant proteins were efficiently transported to mitochondria.

### Recombinant BmANTI1, but not BmANTI2, rescues cellular proliferation caused by BmANTI1 knockdown

BmN4-SID1 cells stably expressing N-terminal 3 x FLAG-tagged BmANTI1 or BmANTI2 ORFs were generated. The distribution of the protein constructs was investigated using subcellular fractionation of cells into cytosolic and mitochondrial compartments. As the anti-α-tubulin antibody efficiently recognizes cytosolic microtubules [44], we were able to use the presence of α-tubulin proteins to verify that the mitochondrial fraction did not include contaminants. An immunoblot analysis showed that BmANTI1 and BmANTI2 were present in both the mitochondrial and cytosolic fractions (Fig. 5D); thus, the recombinant proteins were successfully transported to the mitochondria.

To examine whether expression of BmANTI1 or BmANTI2 could overcome the suppression of cell proliferation in BmANTI1 knockdown cells, we measured cell proliferation rates in BmN4-SID1 cells in which endogenous BmANTI1 was knocked down and which stably expressed BmANTI1 or BmANTI2 constructs. Since *BmAnTI1*-a and *BmANTI1*-b dsRNAs targeted the ORF of the gene, we used *BmANTI1*-UTR dsRNA to ensure silencing only of endogenous BmANTI1 expression. As shown in Fig. 5E, expression of BmANTI1 largely rescued cell proliferation in cells with knockdown of the endogenous BmANTI1; thus, the construct was functional in BmN4-SID1 mitochondria. By contrast, expression of BmANTI2 failed to overcome the inhibition of cell proliferation in BmANTI1 knockdown cells (Fig. 5F). This result is consistent with the hypothesis that BmANTI2 has diverged from BmANTI1 during evolution (Fig. 2) and that the two paralogues are no longer functionally equivalent in silkworms.

### Expression of ANTs from other arthropod species and their cellular distribution

Next, we addressed the question of whether ANTs from other arthropod species could rescue the suppression of cell proliferation following BmANTI1 knockdown. We constructed plasmids expressing one of the following GFP-fused proteins: PxANTI1, PxANTI2, PxANTI3, SgANTI1, SgANTI2, NlANTI1, NlANTI2, DmANT1, or TuANT. The plasmids were transfected into BmN4-SID1 cells. All of the GFP fusion constructs localized to BmN4-SID1 mitochondria in a similar manner as BmANTI1 and BmANTI2 (S1 Fig.). Stable lines expressing these ANTs were generated and fractionated into their cytosolic and mitochondrial compartments. Immunoblot analysis showed that each construct was present in the mitochondria in a similar manner as the BmANT constructs in BmN4-SID1 cells (S2 Fig.).

### Expression of PxANTI1 rescues suppression of cellular proliferation in BmANTI1 knockdown cells

**Fig 6:**
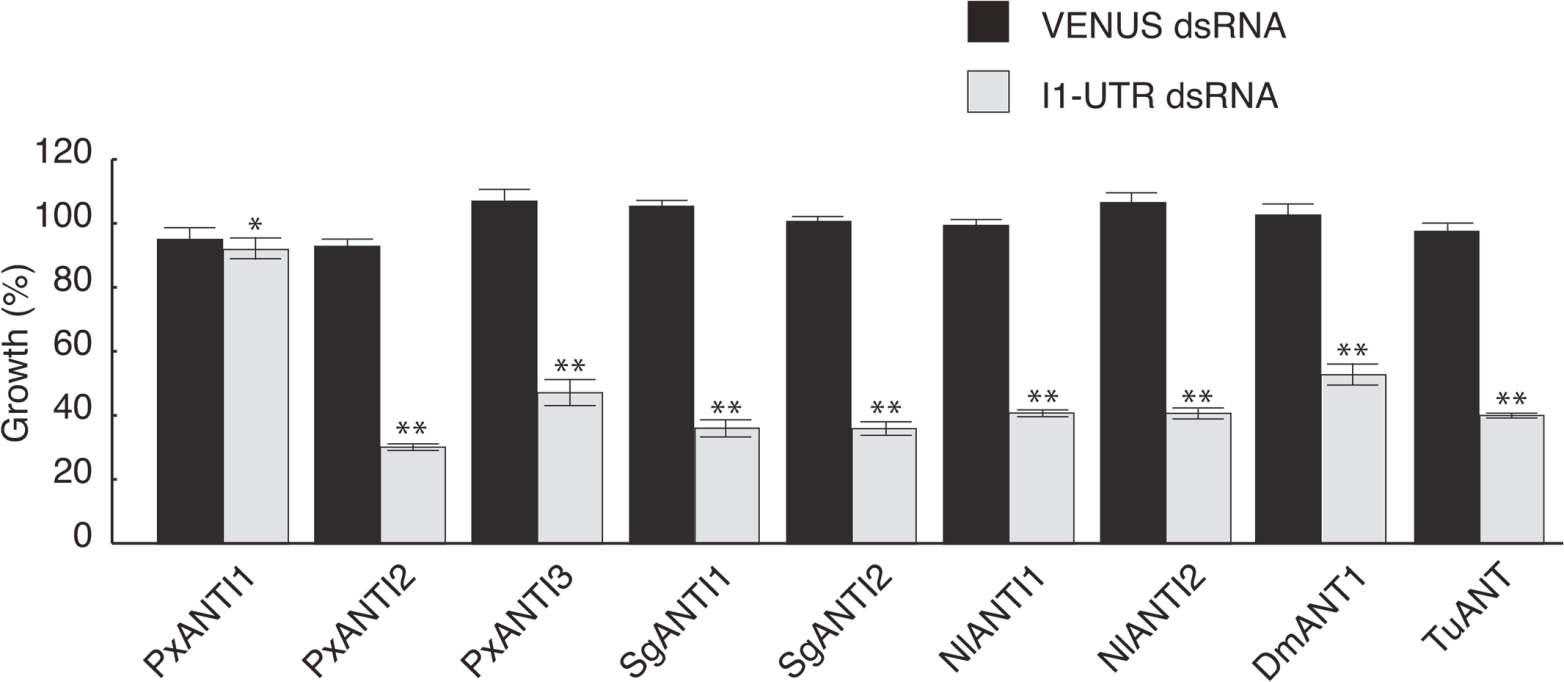
Cell proliferation of BmN4-SID1 cells expressing insect ANTs under knockdown of the endogenous BmANTI1. Decreased cell proliferation of BmN4-SID1 cells silenced the endogenous *BmANTI1* gene can be restored by expression of PxANTI1. Using BmN4-SID1 cells stably expressing FLAG-tagged PxANTI1, PxANTI2, PxANTI3, SgANTI1, SgANTI2, NlANTI1, NlANTI2, DmANT1, or TuANT, cell proliferation assay was performed as described in Fig. 5E. Data are from one of three independent experiments with similar results. Error bars represent the SD values of the means of triplicate wells. Differences in cell proliferation rate between BmANTI1-knockdown cells and cells soaked in VENUS dsRNA were evaluated with a two-tailed Student’s *t*-test. (*P < 0.05; **P < 0.01).

Following knockdown of endogenous BmANTI1 by *BmANTI1*-UTR dsRNA, cell proliferation assays were performed using BmN4-SID1 cells stably expressing PxANTI1, PxANTI2, PxANTI3, SgANTI1, SgANTI2, NlANTI1, NlANTI2, DmANT1, or TuANT (Fig. 6). Only PxANTI1 expression overcame the inhibition of cell proliferation in these cells, indicating that the function of ANTI1 was conserved in *P*. *xylostella*.

### TcANTI2 is required for larval development in *Tribolium*

Our analyses indicated that BmANTI1 and DmANT1 were likely to be homeostatic paralogues of ANT. Next, we investigated whether *Tribolium* had an ANT paralogue that played a similar homeostatic role in beetle larval development. To address whether suppression of TcANT paralogues inhibit larval development, we generated dsRNAs corresponding to the three paralogues and injected them into beetle larvae (S3A Fig.). RT-PCR analysis indicated efficient knockdown of TcANTI1 and TcANTI2, but not TcANTI3 (S3B Fig.). Further analyses showed that silencing of TcANTI1 in *Tribolium* larvae did not affect development, whereas knockdown of TcANTI2 increased the risk of larval lethality and resulted in a low eclosion rate (Table 2). Our results indicate that TcANTI2 is the homeostatic ANT paralogue, whereas TcANTI1 is dispensable for developmental homeostasis.

**Table 2:** Phenotypes of Tribolium larvae depleted for TcANTI1 and TcANTI2 genes.

dsRNA	*n*	Pupation rate (%)	Eclosion rate (%)
*dsVENUS*	49	100	100
*dsTcANTI1-a*	38	100	100
*dsTcANTI1-b*	47	100	100
*dsTcANTI2-a*	50	4	4
*dsTcANTI2-b*	51	0	0

Tribolium larvae were injected the indicated dsRNAs at 14 days after egg laying.

Animals that died within 2 days after injection were eliminated from the analysis.

## Discussion

ANTs are nuclear-encoded mitochondrial proteins that are highly conserved from yeast to mammals. In the present study, we showed that insect ANTs share a common structure composed of three tandem repeats with the consensus sequence PX(D/E)XX(K/R), similar to that seen in vertebrate ANTs (Fig. 1). Like vertebrates, most insects possess more than one ANT paralogue with high sequence similarity, implying that these paralogues are utilized in different cell types depending on external conditions. The silkworm and *Drosophila* genomes encode two ANT paralogues, while the *Tribolium* genome possesses three. BmANTI1, DmANT1, and TcANTI2 appear to play a role in homeostasis in each species. The three ANT paralogues from the genome of the lepidopteran species *P*. *xylostella* were identified by a genome database search and their cDNAs were cloned. The results of our cell proliferation assays suggested that BmANTI1 and PxANTI1 are functional orthologues. PxANTI3, which has a similar amino acid sequence to PxANTI1 (91% identity), may possibly have a function that does not occur in silkworms.

In humans, ANT1 and ANT3 appear to export ATP from mitochondria into the cytosol, whereas ANT2 and ANT4 import ATP into the mitochondria [45]. However, as mentioned in the Introduction, a yeast mutant strain that lacks the three endogenous AAC paralogues can be rescued by expression of each of the HsANTs. It was also previously reported that a single ANT paralogue can specifically exchange ADP/ATP through the mitochondrial inner membrane in both transport directions [46]. The ADP/ATP exchange seems to occur in either direction depending on both the matrix and external ADP/ATP ratios [47]. Thus, the yeast mutant strain might only require the ADP/ATP exchange property, and not any other role of the ANTs. By contrast to the yeast strain, the proliferation deficit in BmANTI1 knockdown cells was not rescued by expression of any insect ANT except for lepidopteran ANTI1. This finding indicates that normal proliferation of the BmANTI1-depleted cells requires not only a protein with an ADP/ATP exchange property, but also one that can perform additional roles such as an interaction with other proteins and/or their post-transcriptional modification.

Since the *ANT4* gene is absent from birds, fish, and frogs, it has been postulated that this paralogue is involved in an energy metabolic pathway that is only present in mammals and reptiles [16]. It is therefore of interest that the similar paralogue to ANT4 is conserved in lepidopteran insects. Moreover, the gene expression profile of *BmANTI2* showed that it has tissue-specific activity. This restricted expression pattern is similar to that identified for *DmANT2* in which a microarray analysis showed testis-specific expression in contrast to the ubiquitous expression of *DmANT1* [30]. Moreover, the report also mentioned that *DmANT1* is very weakly expressed in the testis compared with other tissues, suggesting that *DmANT2* functionally replaces *DmANT1* in this tissue. Thus, DmANT2 is believed to be an orthologue of BmANTI2, suggesting the presence of a similar energy metabolic pathway in mammals, reptiles, Lepidoptera and *Drosophila*.

As mentioned above, ANT2 and ANT4 appear to transport glycolytic ATP toward the mitochondrial matrix under glycolytic conditions; this transport is in the reverse direction to that of ANT1 and ANT3 [45]. The *ANT4* gene is always encoded by autosomes, whereas the *ANT2* gene is located on the X chromosome. In males, genes on the X chromosome are transiently silenced during meiotic prophase (meiotic sex chromosome inactivation) [17,48]. These various lines of evidence have been drawn together in a hypothesis that suggests ANT4 evolved to compensate for the absence of the ANT2 function in spermatocytes [17,49]. With respect to the sex chromosomes, silkworm males are homogametic and have two Z chromosomes. In agreement with a previous report that a significantly higher number of testis-specific genes are present on the Z chromosomes than the autosomes in silkworms [50], *BmANTI2* is located on the Z chromosome while *BmANTI1* is located on an autosome (S2 Table). In contrast to silkworms, *Drosophila* is a male heterogametic species; however, the *Drosophila* X chromosome contains both *DmANT1* and *DmANT2*. In *Drosophila*, meiotic sex chromosome inactivation does not appear to occur in males [51]. In contrast to the chromosomal locations of *DmANT* genes, in *T*. *castaneum* all three *TcANTs* are located on autosomes. Thus, insect *ANTs* may have evolved independently of meiotic sex chromosome inactivation, as has also been suggested for *ANT2* and *ANT4* in anole lizards [16].

Lepidopteran males, including silkworms, produce dimorphic sperm, termed nucleated eupyrene and anucleated apyrene. Apyrene sperm appear to be required for the maturation of the eupyrene sperm [52]. During the larval stage in silkworms, the majority of germ cells develop almost simultaneously in the testis. This synchronicity allowed us to estimate the phase of spermatogenesis in which the *ANT* genes were expressed. Published information indicates that the majority of spermatocytes at the beginning of the 4th instar larval stage are at zygotene and pachytene [53]. We found *BmANTI2* expression in the testis of larvae at this stage, implying a role in supplying a large amount of ATP to spermatocytes during the early stages of meiotic prophase I. This interpretation is consistent with the severe disruption of the seminiferous epithelium in germ cells of *Ant4*-deficient mice [18]. At the beginning of the 5th instar larval stage in silkworms, spermatids appear in the testis; these spermatids subsequently start to mature into fully formed eupyrene spermatozoa at the spinning stage of larval development. *BmANTI2* transcript levels increased from the 4th instar stage to the spinning stage (Fig. 3C).

Our observation of an increase in transcript levels raises the question of whether this was associated with the number and size of spermatocytes or spermatids. During the 4th instar larval stage, the number of spermatocytes is essentially constant since most germ cells in the testis are at meiotic prophase I [53]. As the germ cells progress through meiosis, the number of spermatocytes and spermatids will increase from day 1 of the 5th instar larval stage. Electron microscopic analysis shows that eupyrene spermatocytes increase in volume during prophase I [54]. At pachytene, the volume of mitochondria is significantly increased in eupyrene spermatocytes but not apyrene spermatocytes [54]. During elongation of eupyrene spermatids, the mitochondria swell without reducing the high density of the mitochondrial DNA. Thus, the increased levels of *BmANTI2* transcripts may reflect the increase in the mitochondria in eupyrene spermatocytes and spermatids.

Silkworm spermatozoa have a unique energy metabolic pathway involving extracellular glycolysis activated by a serine endopeptidase known as initiatorin [55]. This pathway includes an arginine degradation cascade, which has also been observed in *Drosophila* [56], suggesting that the glycolysis pathway is conserved in these species. The similar expression profiles and the N-terminal extensions of BmANTI2 and DmANT2 may indicate the involvement of these proteins in a common mechanism, such as the extracellular glycolysis pathway. In addition to the BmANTI2 protein, the silkworm testis appears to contain testis-specific mitochondrial paralogues such as ATPase inhibitor-like protein-b [57] and some members of the mitochondrial carrier protein family (manuscript in preparation). The presence of these proteins suggests that the components of the mitochondrial inner membrane may differ partially in sperm and eggs. Whether human spermatozoa possess a similar extracellular glycolytic pathway remains to be determined. Further investigation of these testis-specific mitochondrial paralogues, such as BmANTI2 and ATPase inhibitor-like protein-b, will undoubtedly lead to a greater understanding of the molecular mechanism of energy metabolism in spermatozoa bioenergetics.

## Supporting Information

S1 Fig Subcellular localization of GFP-fused insect or mite Ants in BmN4-SID1 cells. Each GFP-fused PxANTI1, PxANTI2, PxANTI3, SgANTI1, SgANTI2, NlANTI1, NlANTI2, DmANT1 or TuANT was transiently expressed in BmN4-SID1 cells. The subcellular localizations of these constructs were observed as described in Fig. 5C.
(EPS)

S2 Fig Cell distribution of FLAG-tagged insect or mite Ants in BmN4-SID1 cells. FLAG-tagged (A) PxANTI1, PxANTI2, PxANTI3, (B) SgANTI1, SgANTI2, (C) NlANTI1, NlANTI2, (D) DmANT1, and (E) TuANT were stably expressed in BmN4-SID1 cells, and the cells were fractionated into cytosolic (Cyto) and mitochondrial (Mito) fractions. Whole-cell lysates (WCL) were included to confirm protein expression. Each fraction was immunoblotted with anti-FLAG M2 and anti-α-tubulin antibodies.
(EPS)

S3 Fig Verification of gene silencing of TcANTs mediated by dsRNAs. (A) Lines on a schematic diagram of TcANTI1-3 represent relative positions of dsRNA-targeted regions. The length of dsRNAs is shown in parentheses. (B) The indicated dsRNAs were injected into larvae, following which the insects were raised individually in 24-well microtiter plates with whole wheat flour at 30°C. Three were sampled at 3 days after injection for each treatment and subjected to semi-qRT-PCR analysis. Transcript for *Tribolium* ribosomal protein 6 (TcrpS6) serves as an internal control.
(EPS)

S1 Table Primer sequences of Insect *ANTs* and control genes. List of primers used in this study.
(EPS)

S2 Table Chromosomal locations of the ANT genes in *D*. *melanogaster*, *B*. *mori*, and *T*. *castaneum*. Chromosomal locations of the DmANTs, BmANTs, and TcANTs were examined using the databases of FlyBase, KAIKObase, and Beetlebase.
(EPS)
